# Substitution of Zn^2+^ with Ni^2+^ Alters Kinetic Steps in D‑2-Hydroxyglutarate Dehydrogenase from *Pseudomonas aeruginosa* PAO1

**DOI:** 10.1021/acsomega.5c09933

**Published:** 2025-12-15

**Authors:** Bilkis Mehrin Moni, Junkai Yang, Joanna Afokai Quaye, Giovanni Gadda

**Affiliations:** †Departments of Chemistry, ‡Biology, and §The Center for Diagnostics and Therapeutics, 1373 Georgia State University, Atlanta, Georgia 30302-3965, United States

## Abstract

D-2-hydroxyglutarate dehydrogenase from *Pseudomonas aeruginosa* PAO1 (EC:1.1.99.39; UniProt ID: Q9I6H4) is an FAD- and Zn^2+^-dependent metallo-flavoenzyme (E_FAD_-Zn^2+^) that catalyzes D-2-hydroxyglutarate oxidation to 2-ketoglutarate, playing a crucial role in l-serine biosynthesis and serving as a potential therapeutic target against the bacterium. The enzyme is also active with the alternative metal ion Ni^2+^. However, the effects of substituting Zn^2+^ with the more electronegative and smaller ionic radius Ni^2+^ (E_FAD_-Ni^2+^) on enzyme function remain unexplored. This study utilized steady-state kinetics, rapid reaction kinetics, and kinetic solvent viscosity analysis to investigate the effects of metal ion substitution on the catalytic steps of the enzyme. The kinetic solvent viscosity effects on the *k*_cat_ parameter indicate that the flavin reduction and product release limit the overall catalytic turnover of E_FAD_-Zn^2+^, with slopes of 0.52. In contrast, E_FAD_-Ni^2+^ exhibits no viscosity effects on the *k*_cat_ and *k*_cat_/*K*_m_ parameters, indicating rapid substrate dissociation and product release and that the turnover is not limited by product release. The first-order rate constant for flavin reduction at a saturating D-malate concentration, *k*_red_, which reports the hydride transfer reaction, was comparable between E_FAD_-Ni^2+^ (*k*_red_ = 84 s^–1^) and E_FAD_-Zn^2+^ (*k*_red_ = 68 s^–1^). These findings provide mechanistic insight into how replacing Zn^2+^ with Ni^2+^ alters the rate-limiting steps in D-2-hydroxyglutarate dehydrogenase. The study also highlights how the physicochemical properties of the metal cofactors, such as electronegativity and ionic radius, can influence the catalytic steps of the enzyme including substrate and product release.

## Graphical abstract

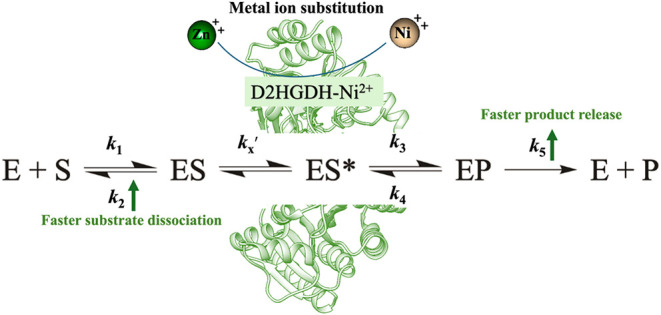

## Introduction

Metalloenzymes substituted with alternative metal ions often display significant variations in catalytic activity, even when the substituted ions exhibit broadly similar chemical properties to the native ones, including comparable ionic radius, ionic charge, size, electronegativity, mass, redox potential, electronic configuration, and preferred coordination geometry.^1–14^ The fact that metalloenzymes often exhibit dramatic changes in their catalytic activity when subjected to chemically similar but non-native metal substitutions is a long-standing puzzle.^1,2,8,12,15–17^ A classical example of metalloenzymes is human carbonic anhydrase,^8,18–20^ one of the earliest known zinc metalloenzymes, which shows significant alterations in the enzyme catalytic activity when zinc is replaced with other transition metal ions Co^2+^, Ni^2+^, Cu^2+^, Cd^2+^, and Mn^2+^.^8,18–20^ Similar findings have been reported for several other zinc-dependent metalloenzymes, including astacin,^17,21–23^ carboxypeptidase A,^24–27^ and thermolysin,^28–31^ where metal substitution has been used to probe the structural and mechanistic roles of metal ions in catalysis.^25,29,31^ These studies have underscored the metal substitution tolerance of the enzymes, yet the dramatic and sometimes unpredictable shifts in catalytic behavior.

A subset of flavin-containing enzymes are known to bind metal ions as cofactors, forming a unique class referred to as metallo-flavoenzyme.^32–41^ Studies on several of these flavin-dependent metalloenzymes, including *Methanobacterium formicicum* formate dehydrogenase, mammalian histone demethylase, D-lactate dehydrogenase, avian sulfhydryl oxidase, and various D-2-hydroxyglutarate dehydrogenases homologues (D2HGDHs), have shown that metal ions such as Zn^2+^, Ni^2+^, Co^2+^, Mn^2+^, Cd^2+^, and Fe^2+^ can either enhance or inhibit enzymatic activity.^35–45^ However, the impact of substituting the native-bound metal ion with an alternative metal ion on the kinetic mechanism of metallo-flavoenzymes remains largely unexplored.

*Pseudomonas aeruginosa* D-2-hydroxyglutarate dehydrogenase (D2HGDH; EC 1.1.99.39; UniProt ID: Q9I6H4) is a recently characterized FAD- and Zn^2+^-dependent metallo-flavoenzyme (Figure 1),^46–49^ belonging to the PF01565 and PF02913 superfamilies of FAD-dependent enzymes.^50^ *P. aeruginosa* D2HGDH catalyzes the oxidation of D-2-hydroxyglutarate (D2HG) to the TCA cycle intermediate 2-ketoglutarate, supporting l-serine biosynthesis and bacterial survival (Scheme 1).^37,38,46–52^ Since the enzyme’s initial characterization in 2020, the enzyme has emerged as a model system for exploring flavoenzymes that require metal cofactors in the active sites.^49,50^ Prior to 2023, it was unclear whether D2HGDH enzymes required metal ions as essential cofactors or merely as activators.^46,49^ Multiple reports on D2HGDH homologues suggested that enzymatic activity could either increase or decrease in the presence of metal ions.^49,53^ Among these, Zn^2+^ consistently stood out as the ion most commonly associated with activity enhancement of the enzymes.^49^ However, the 2023 study on *P. aeruginosa* D2HGDH provided the first clear evidence that Zn^2+^ is an essential cofactor for catalysis.^46^ Zn^2+^ was the only metal ion detected in significant amounts upon metal component analysis, and its presence correlated directly with enzymatic function.^46^ Furthermore, Zn^2+^ was found occupying the metal-binding site in the only available crystal structure of a D2HGDH homologue, from *Homo sapiens*.^54–56^

**Figure 1:**
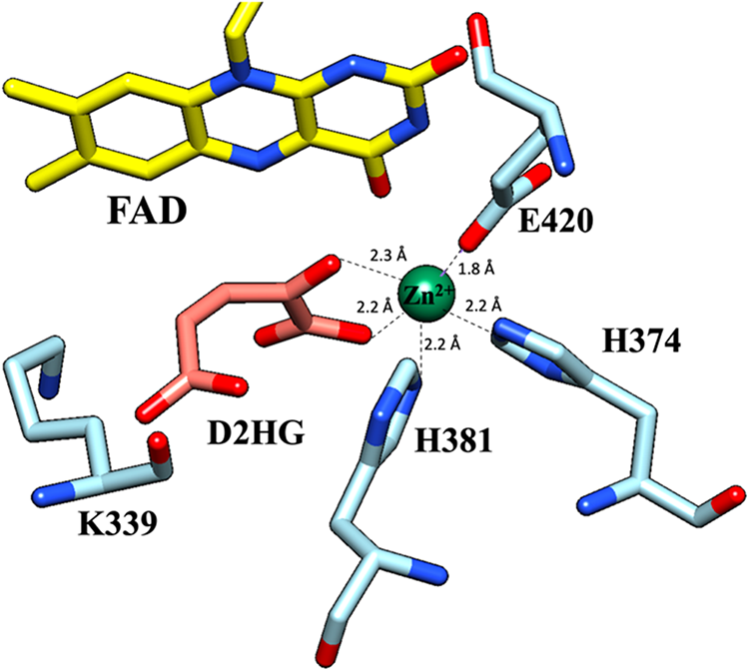
Active-site topology of *P. aeruginosa* D2HGDH. The active site is depicted with the enzyme bound to substrate D2HG and the conserved active site residues involved in Zn^2+^ coordination. The enzyme model was built using SWISS-MODEL with a putative dehydrogenase from *Rhodopseudomonas palustris* (Protein Data Bank code: 3PM9) as a template, yielding a structure similar to that of the human D2HGDH crystal structure. The substrate D2HG and Zn^2+^ metal were obtained by the structural overlay of the *P. aeruginosa* enzyme and its human homologue (PDB: 6LPP). The isoalloxazine ring of the FAD cofactor is shown in yellow; the Zn^2+^ cofactor is shown as a green sphere; the substrate D2HG is shown in orange; the active-site residues are shown in cyan; nitrogen atoms are shown in blue; and oxygen atoms are shown in red. The protein structures were visualized using UCSF Chimera.^57^

**Scheme 1:**
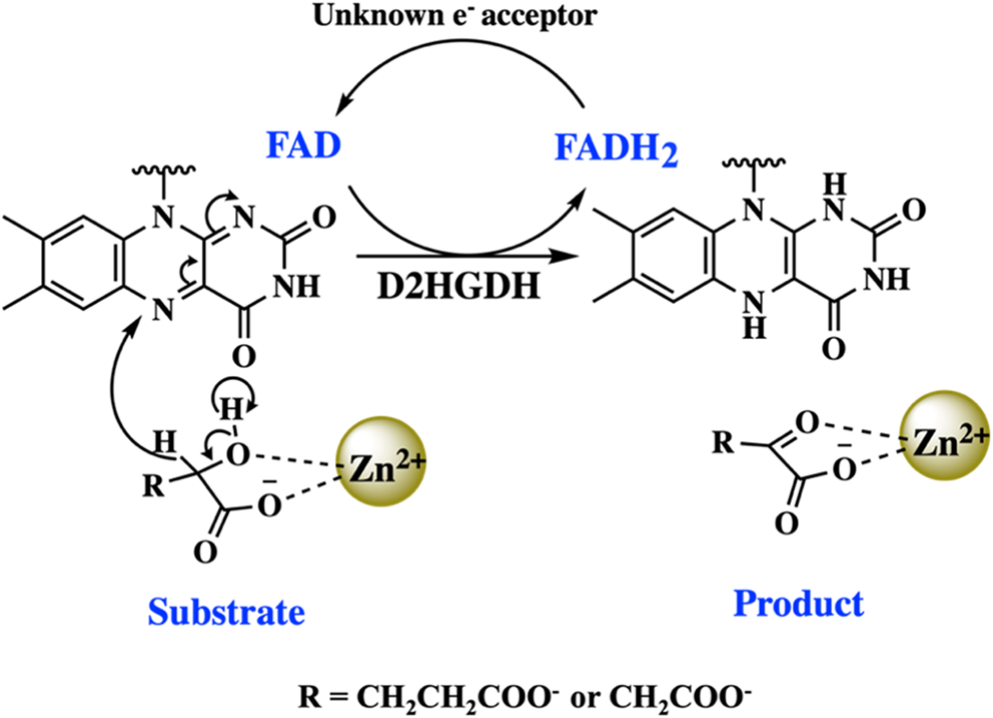
Reaction Mechanism of D-2-Hydroxyglutarate Dehydrogenase with D-2-Hydroxyglutarate or D-Malate as a Substrate^a^

a D2HGDH, D-2-hydroxyglutarate dehydrogenase.

Although Zn^2+^ is the native cofactor, *P. aeruginosa* D2HGDH also retains catalytic activity in the presence of alternative metal ions such as Co^2+^, Mn^2+^, Ni^2+^, and Cd^2+^.^46,47,49,58,59^ Among these, Ni^2+^, despite its smaller ionic radius and higher electronegativity, can substitute for Zn^2+^ without compromising flavin integrity or the overall structural fold of the enzyme.^47,58,59^ However, the functional consequences of metal ion substitution on specific catalytic steps of *P. aeruginosa* D2HGDH, particularly substrate binding, product release, and overall rate-limiting steps, remain unexplored. Previous studies have proposed a mechanistic model for *P. aeruginosa* D2HGDH, implicating a metal ion Zn^2+^ as the proton abstractor from C2 hydroxyl groups of the α-hydroxy acid substrate that initiates hydride transfer to FAD.^48,49^ While the histidine-based model is standard in FMN-dependent α-hydroxy acid oxidizing enzymes,^60–67^ recent evidence points to a more direct catalytic role for the Zn^2+^ in *P. aeruginosa* D2HGDH.^48,49^ Furthermore, the sequence of metal ion and substrate binding appears critical for catalysis, with flavin reduction occurring only when Zn^2+^ is prebound to the enzyme before substrate addition.^46–49,58^ Despite these insights, it is still unclear how substitution with a non-native metal ion like Ni^2+^ affects the enzyme’s kinetic mechanism. Does Ni^2+^ merely restore activity, or does it alter the fundamental catalytic profile of the *P. aeruginosa* D2HGDH?

In this study, we investigated how replacing Zn^2+^ with Ni^2+^ in *P. aeruginosa* D2HGDH affects the individual steps of the catalytic cycle of the enzyme. Using steady-state kinetics, rapid reaction kinetics, and solvent kinetic viscosity effects, we examined the influence of Ni^2+^ on substrate binding, flavin reduction, and product release of the *P. aeruginosa* D2HGDH. Our goal was to determine whether the distinct physicochemical properties of Ni^2+^, namely, the higher electronegativity and smaller ionic radius, affect the rate-limiting steps and mechanistic behavior of the metallo-flavoenzyme. These findings provide new insights into how alternative metal cofactors shape enzyme catalysis and may inform future efforts in metallo-flavoenzyme engineering and the development of antibacterial drugs against *P. aeruginosa*.

## Materials and Methods

### Materials

The pET20b­(+) plasmid harboring the 6 His-tag at the tail of the gene for *P. aeruginosa* D-2-hydroxyglutarate dehydrogenase was designed in the lab and purchased from GenScript (Piscataway, NJ). *Escherichia coli* strain Rosetta­(DE3)­pLysS was from Novagen (Madison, WI). Bovine serum albumin (BSA) was purchased from Promega (Madison, WI). Luria–Bertani agar, Luria–Bertani broth, chloramphenicol, IPTG, lysozyme, sodium hydrosulfite (dithionite), phenazine methosulfate (PMS), and PMSF were obtained from Sigma-Aldrich (St. Louis, MO). Ampicillin was purchased from ICN Biomedicals (Aurora, OH). D-2-Hydroxyglutarate was purchased from Millipore Sigma (Burlington, MA). D-Malate was purchased from Alfa Aesar (Haverhill, MA). Zinc chloride and nickel chloride were purchased from Sigma-Aldrich (St. Louis, MO). Glucose, glycerol, and all other commercially available reagents were of high purity.

### Protein Purification and Metal Component Analysis

To obtain pure His-tagged D-2-hydroxyglutarate dehydrogenase loaded with Zn^2+^ or Ni^2+^, for kinetic studies, the enzyme species were expressed in *E. coli* strain Rosetta­(DE3)­pLysS and purified, following the described protocol.^46,47,58,59^ The concentration of the total enzyme as purified was determined using the Bradford method.^68^ The concentration of the FAD-bound enzyme was estimated from the FAD absorbance at 450 nm using an experimentally determined ε_450_ value of 12,500 M^–1^ cm^–1^. The metal composition was analyzed by using inductively coupled plasma-mass spectrometry (ICP-MS) to determine the concentrations of metal ions in the purified enzyme species. Aliquots of each enzyme species were dialyzed against 2 L of deionized water for 24 h to remove all unbound metal ions. The resulting enzymes were then sent for ICP-MS analyses at the Center for Applied Isotope Studies (CAIS), University of Georgia, Athens, GA. The resulting data were analyzed using Microsoft Excel.

### Kinetic Assays

Enzyme activity was measured polarographically by monitoring the rate of PMS-driven oxygen consumption using a Hansatech oxygen electrode, which was thermostated at 25 °C. The determination of the steady-state kinetic parameters was carried out at varying concentrations of D-malate or D-2-hydroxyglutarate (D2HG) and a fixed saturating concentration of 1 mM PMS as an artificial electron acceptor by measuring initial reaction rates for each enzyme in 25 mM NaPO_4_, pH 7.4, at 25 °C. In this assay, PMS transfers electrons from the reduced flavin to molecular oxygen, allowing oxygen consumption to be monitored as an indirect measure of dehydrogenase activity.^69^ The concentration ranges for D-malate were 1–60 mM for E_FAD_-Zn^2+^ and 0.08–15 mM for E_FAD_-Ni^2+^. The concentration of D2HG was 0.01–1 mM for both enzyme species, and the PMS concentration was fixed at 1 mM. To determine the saturating concentration of PMS, the steady-state kinetic parameters of the enzyme were measured at three fixed PMS concentrations (0.1, 1, and 2 mM) while varying the concentration of D-malate.

The anaerobic reduction of the metal-ion-loaded enzyme species E_FAD_-Zn^2+^ and E_FAD_-Ni^2+^ with D-malate in 25 mM NaPO_4_, pH 7.4, was monitored using an SF-61DX2 Hi-Tech KinetAssyst high-performance stopped-flow spectrophotometer (Bradford-on-Avon, U.K.), which was maintained at 25 °C with a thermostated water bath. Anaerobiosis of the instrument and all buffers, substrates, and enzyme solutions was performed according to the standard procedure.^48,70,71^ After mixing, the enzyme concentration was ∼10 μM and D-malate ranged from 1 to 60 mM for E_FAD_-Zn^2+^, and 0.3 to 25 mM for E_FAD_-Ni^2+^ to maintain pseudo-first-order conditions.

To investigate the effects of solvent viscosity on the kinetic properties of the different metal ions loaded enzyme species, E_FAD_-Zn^2+^ and E_FAD_-Ni^2+^, the steady-state kinetic parameters were determined by varying the concentration of D-malate (0.5–25 mM for E_FAD_-Zn^2+^, and 0.25–50 mM for E_FAD_-Ni^2+^), with a reaction buffer of 25 mM NaPO_4_, pH 7.4 and 25 °C, containing varying amounts of glycerol (0–40%, m/m, η_rel_ = 1.0–3.5 cP). Assay reaction mixtures were equilibrated at atmospheric oxygen for at least 2 min before the reaction was initiated with the addition of the enzyme. The experiment was repeated using glucose as a viscosigen (0 – 34%, m/m, η_rel_ = 1.0–3.6 cP) for both enzyme species. The relative viscosities of all viscosigens were comparable.

### Data Analysis

The initial rates of oxygen consumption were observed with a computer-interfaced Oxy-32 oxygen-monitoring system (Hansatech Instruments Ltd., Norfolk, U.K.). The steady-state kinetic parameters for the enzymatic assay were obtained by fitting the experimental data points to the Michaelis–Menten equation (eq 1) using Kaleida Graph software (Synergy Software, Reading, PA).

1 $$v_{o}/e=\frac{k_{\mathrm{cat}}\left[A\right]}{K_{m}+\left[A\right]}$$

In the above equation, *v*_*o*_ is the initial velocity, *e* represents the enzyme concentration and *k*_cat_ is the turnover rate at a saturating concentration of both substrates.

Stopped-flow traces obtained with the KinetAsyst 3 (TgK-Scientific, Bradford on-Avon, U.K.) software were fitted to eq 2, which describes a single-exponential process.

2 $$A=B_{1}e^{-k}\mathrm{obs}1^{t}+C$$

In this equation, *A* represents the absorbance at 450 nm at time *t*; *B*_1_ represents the amplitudes of the decrease in absorbance; and *k*_obs1_ defines the observed rate constants for the change in absorbance. *C* is an offset value that accounts for the nonzero absorbance of the enzyme-bound reduced flavin at an infinite time.

The concentration dependence for the observed rate constants of flavin reduction was analyzed with eq 3, where *S* represents the concentration of D-malate, *k*_red_ is the rate of flavin reduction at a saturating concentration of D-malate, *k*_rev_ is the reverse rate of enzyme catalysis that reports on the conversion of the enzyme–product complex (EP) to the enzyme–substrate complex (ES), and *K*_d_ is the dissociation constant for D-malate binding.

3 $$k_{\mathrm{obs}}=\frac{k_{\mathrm{red}}\;s}{k_{d}+s}+k_{\mathrm{rev}}$$

The data from the viscosity effects on the *k*_cat_ and *k*_cat_/*K*_m_ values were fit to eq 4, where (kinetic parameter)_o_ and (kinetic parameter)_η_ are the values for the kinetic parameters in the absence and presence of viscosigen, respectively. *S* is the degree of viscosity dependence, and η_rel_ is the relative viscosity of the buffered solution.

4 $$\frac{{\left(kinetic\;parameter\right)}_{o}}{{\left(kinetic\;parameter\right)}_{\eta }}=S\left(\eta_{rel}-1\right)+1$$

## Results

### Protein Purification and Metal Component Analysis of E_FAD_-Ni^2+^

The E_FAD_-Ni^2+^ enzyme was expressed in *E. coli* strain Rosetta­(DE3)­pLysS and purified to electrophoretic homogeneity, as confirmed by sodium dodecyl sulfate–polyacrylamide gel electrophoresis (SDS–PAGE) analysis showing a single prominent band corresponding to a molecular weight of ∼51.3 kDa (Figure S1). The enzyme was purified following the same protocol previously used for the E_FAD_-Zn^2+^ enzyme.^46–48,58^ Typically, ∼110 mg of purified enzyme was obtained from a 2 L cell culture. The purified E_FAD_-Ni^2+^ enzyme was in a fully oxidized state with the bound FAD cofactor, as indicated by the ultraviolet–visible (UV–vis) absorbance spectrum showing absorbance maxima at 380 and 450 nm (data not shown). Inductively coupled plasma-mass spectrometry (ICP-MS) was employed to determine the incorporation of metal ions into the purified enzyme species. When 1 mM chloride salts of the metal ions were present in the purification buffers, the observed mol Ni^2+^ to mol protein ratio was 1.8:1 for E_FAD_-Ni^2+^, which was comparable to that of E_FAD_-Zn^2+^ with a mol Zn^2+^ to mol protein ratio of 2.2:1 (Table 1), due to the presence of two distinct metal-binding sites comprising a high-affinity catalytic site, and a low-affinity nonspecific metal-binding site as previously determined for the E_FAD_-Zn^2+^.^58^ Additionally, nonstoichiometric, yet significant, amounts of the noncatalytic metal ion Mg^2+^ were detected in both enzyme species (Table 1). These results confirm the successful incorporation of the respective metal ions into the purified enzyme species.

**Table 1:** Metal Composition of the Different Forms of D-2-Hydroxyglutarate Dehydrogenase^a^

	^b^[M]_E‑Zn_^2+^, μM	^c^[M]_E‑Ni_^2+^, μM
	(M^2+^: E)^47^	(M^2+^: E)
M^2+^	420 (2.2:1)^47^	88 (1.8:1)
Mg^2+^	39 (0.2:1)^47^	11 (0.2:1)

a E: D2HGDH; M^2+^: Metal (Zn^2+^, or Ni^2+^).

b 195 μM protein.^47^

c 50 μM protein. ≤5% standard error was recorded for all numbers.

### Steady-State Kinetic

The steady-state kinetic parameters of E_FAD_-Ni^2+^ were determined and compared to those of E_FAD_-Zn^2+^ to investigate how the substitution of the native bound to the alternative metal ions affects the catalytic turnover and efficiency of D-2-hydroxyglutarate dehydrogenase. The kinetic parameters of the E_FAD_-Ni^2+^ were determined by measuring the initial rate of oxygen consumption at varying concentrations of the physiological substrate D2HG or the alternative substrate D-malate and fixed saturating 1 mM PMS in 25 mM NaPO_4_, pH 7.4, and 25 °C, and compared to those of E_FAD_-Zn^2+^ (Tables 2 and 3). PMS at 1 mM was confirmed to be saturating for both E_FAD_-Zn^2+^ and E_FAD_-Ni^2+^, as kinetic parameters obtained at 1 and 2 mM PMS were comparable across a range of D-malate concentrations (Table S1). The Michaelis–Menten equation (eq 1) was used to obtain the best fit for the kinetic data of both E_FAD_-Ni^2+^ and E_FAD_-Zn^2+^, based on the initial reaction rates with various substrate concentrations. With the physiological substrate D2HG, there was a 2-fold decrease in the *k*_cat_ value and a 1.5-fold decrease in the *k*_cat_/*K*_m_ value, while the *K*_m_ value was almost similar in both enzymes upon metal substitution from Zn^2+^ to Ni^2+^ (Table 2). Similarly, when the alternative substrate D-malate was tested, the data showed that E_FAD_-Ni^2+^ catalyzes the oxidation of D-malate to oxaloacetate with only a 4-fold decrease in the *k*_cat_ value, a 2.4-fold increase in the *k*_cat_/*K*_m_ value, and a 10-fold decrease in the *K*_m_ value relative to that of E_FAD_-Zn^2+^ (Table 3). Despite differences in the *K*_m_ values across the two enzymes, a direct interpretation cannot be provided in this study, as the *K*_m_ values reflect different steps in the kinetic schemes of E_FAD_-Ni^2+^ and E_FAD_-Zn^2+^. Hence, the comparison of the *K*_m_ values across both enzymes is limited to the numerical difference observed herein. A derivation for the *K*_m_ parameter in both enzymes is provided in **Footnote**.[Fn fn1]

**Table 2:** Steady-State Kinetics of D-2-Hydroxyglutarate Dehydrogenase Enzyme Species with Varying D2HG and Fixed Saturating PMS^a^

enzymes	*k*_cat_, s^–1^	*K*_m_, mM	*k*_cat_/*K*_m_, M^–1^s^–1^
E_FAD_-Zn^2+^	30 ± 1	0.15 ± 0.01	205,000 ± 10,000
E_FAD_-Ni^2+^	18 ± 1	0.14 ± 0.01	130,000 ± 9000

a The kinetic parameters were determined with 1 mM fixed saturating PMS in 25 mM NaPO_4_, pH 7.4 at 25 °C. Standard errors are from individual fits of the kinetic data.

**Table 3:** Steady-State Kinetics of D-2-Hydroxyglutarate Dehydrogenase Enzyme Species with Varying D-Malate and Fixed Saturating PMS^a^

enzymes	*k*_cat_, s^–1^	*K*_m_, mM	*k*_cat_/*K*_m_, M^–1^s^–1^
E_FAD_-Zn^2+^	38 ± 1	3.8 ± 0.7	10,000 ± 1,000
E_FAD_-Ni^2+^	10 ± 1	0.4 ± 0.03	24,000 ± 2000

a The kinetic parameters were determined with 1 mM fixed saturating PMS in 25 mM NaPO_4_, pH 7.4 at 25 °C. Standard errors are from individual fits of the kinetic data.

D-malate was selected as a cost-effective model substrate for further kinetic studies since its catalytic turnover is comparable to that of D2HG.

### Reductive Half-Reaction with D-Malate

As the first step toward the mechanistic characterization of D-malate oxidation by E_FAD_-Ni^2+^ enzyme and compared with those of previously published E_FAD_-Zn^2+^,^47^ the rate of flavin reduction was determined by anaerobic mixing of the E_FAD_-Ni^2+^ enzyme with D-malate by monitoring the rates of decrease in absorbance at 450 nm as a function of the concentration of the substrate in a stopped-flow spectrophotometer in 25 mM NaPO_4_ at pH 7.4 (Figure 2). As illustrated in the example of Figure 2A, the E_FAD_-Ni^2+^ enzyme-bound flavin was reduced to the hydroquinone state in a monophasic pattern without detection of transient species, with a *k*_red_ value of 84 s^–1^, a *k*_rev_ value of 9 s^–1^, and a *K*_d_ value of 4 mM (Table 4). In contrast, a biphasic reduction of the flavin was observed in the E_FAD_-Zn^2+^ enzyme,^47^ with a *k*_red_ value of 68 s^–1^, *k*_rev_ value of 4 s^–1^, and a *K*_d_ value of 8 mM (Table 4). The fast phase accounted for more than 90% of the total absorbance change at 450 nm and was attributed to flavin reduction.^47^ The slow phase (∼10% of the total absorbance change, and a substrate-concentration-independent *k*_obs_ value of ∼3 s^–1^) likely arose from a nonfunctional enzyme generated during anaerobic sample preparation.^47^

**Figure 2:**
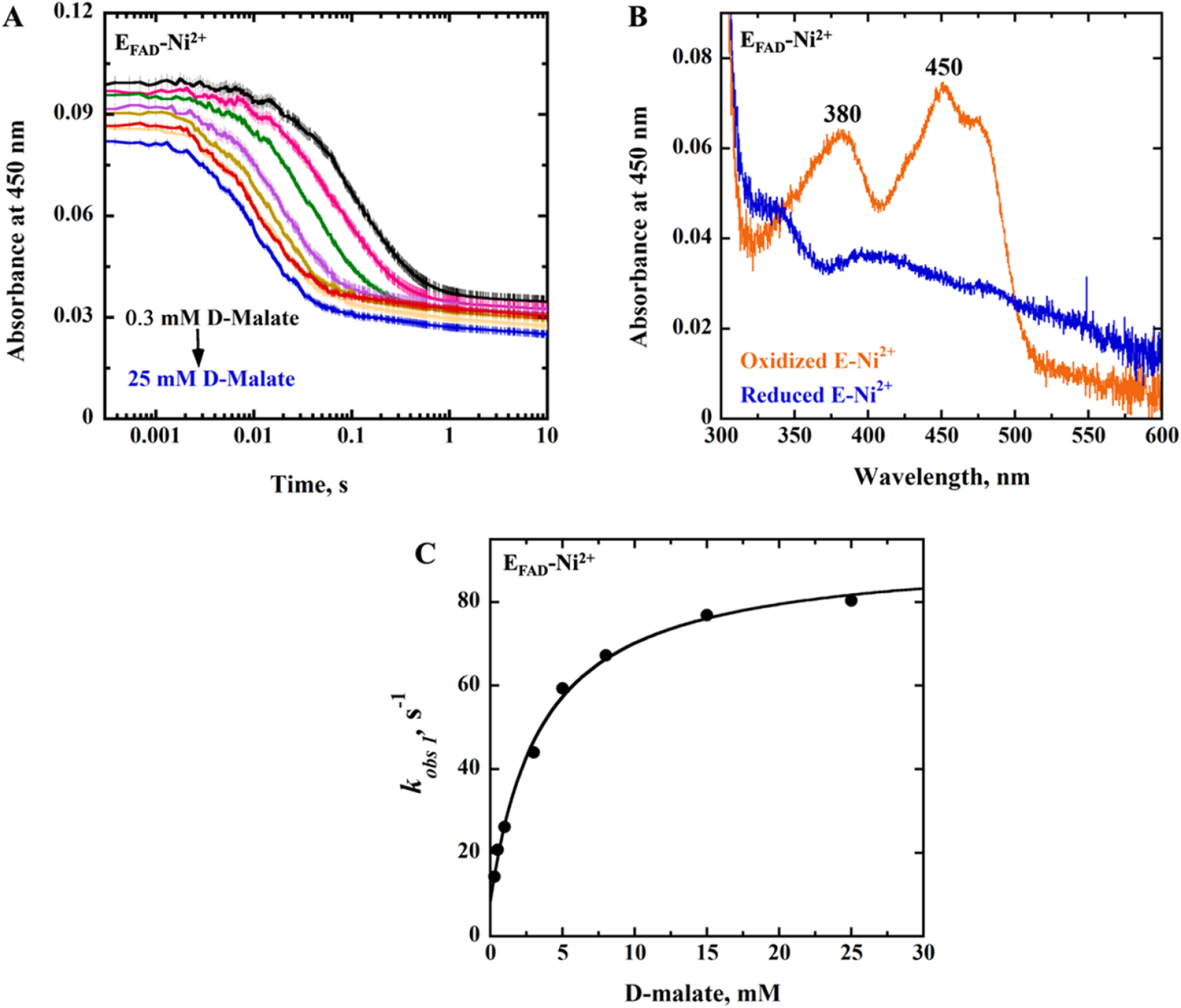
Anaerobic reduction of E_FAD_-Ni^2+^ with D-malate. (A) Stopped-flow traces of E_FAD_-Ni^2+^ at 450 nm with varying concentrations of D-malate (0.3–25 mM) fit eq 2. Note the log time scale. For clarity, one out of every 10 experimental points is shown (vertical lines). The instrument dead time is 2.2 ms. (B) Absorption spectra of E_FAD_-Ni^2+^ showing the fully oxidized flavin before reduction (orange trace) and fully reduced flavin after reduction with 0.3 mM D-malate (blue trace). (C) Concentration dependence of the observed rate constant (*k*_obs1_) for flavin reduction of E_FAD_-Ni^2+^ with D-malate, fit to eq 3. The experiment was carried out in 25 mM NaPO_4_, pH 7.4, using an SF-61DX2 Hi-Tech KinetAsyst high-performance stopped-flow spectrophotometer, thermostated at 25 °C, and equipped with a photomultiplier tube detector under anaerobic conditions.

**Table 4:** Reductive Half-Reaction of D-2-Hydroxyglutarate Dehydrogenase Enzyme Species with Substrate D-Malate^a^

enzymes	*k*_red_, s^–1^	*K*_d_, mM	*k*_rev_, s^–1^
E_FAD_-Zn^2+^	68 ± 2	8 ± 1	4 ± 2
E_FAD_-Ni^2+^	84 ± 2	4 ± 1	9 ± 2

a The rapid reaction kinetic parameters were determined using D-malate in 25 mM NaPO_4_, pH 7.4, at 25 °C. Standard errors are from individual fits of the kinetic data.

### Kinetic Solvent Viscosity Effects

A previous study on D-2-hydroxyglutarate dehydrogenase showed that the hydride transfer and product release are partially rate-limiting for the overall turnover of the E_FAD_-Zn^2+^ complex with D-malate.^47^ To determine the effect of alternative metal ion Ni^2+^ on the relevant kinetic steps during the enzyme turnover, kinetic solvent viscosity effects (KSVE) were measured on the steady-state kinetic parameters of the E_FAD_-Ni^2+^ with D-malate at varying concentrations of glycerol or glucose as an added viscosigen at pH 7.4 and 25 °C and compared to those of E_FAD_-Zn^2+^ (Table 5, Figures 3 and 4). The effect of solvent viscosity on the *k*_cat_ and *k*_cat_/*K*_m_ values for E_FAD_-Ni^2+^ was investigated to probe whether kinetic steps of substrate binding and product release were at least partially rate-limiting for the reductive half-reaction in which the D-malate is oxidized to oxaloacetate and the overall turnover of the enzyme. A plot of normalized *k*_cat_ versus relative viscosity of the solvent for E_FAD_-Ni^2+^ yielded a slope of zero, consistent with the rate of product release having a minimal impact on the overall turnover of the enzyme with D-malate (Table 5, Figures 3 and 4). Similarly, a slope of ∼0.10 was determined in a plot of normalized *k*_cat_/*K*_m_ value versus relative viscosity of the solvent, suggesting that kinetic steps of substrate binding do not limit the rate of the reductive half-reaction in E_FAD_-Ni^2+^ (Table 5, Figures 3 and 4).

**Figure 3:**
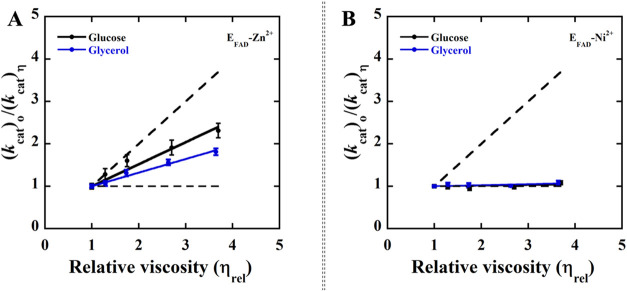
Effects of solvent viscosity on the *k*_cat_ parameters with the substrate D-malate. (A) Viscosity effects on the *k*_cat_ parameter of E_FAD_-Zn^2+^. (B) Viscosity effects on the *k*_cat_ parameter of E_FAD_-Ni^2+^. The dashed line with a slope of 1 and a slope of 0 describes a case in which the reaction is diffusion-controlled and not affected by diffusion, respectively. The enzymes activity assays were carried out using a Clark-type oxygen electrode in 25 mM NaPO_4_, 1 mM PMS, pH 7.4 thermostated at 25 °C, containing varying amounts of glycerol: blue (0–40%, m/m, η = 1.0–3.5 cP) and glucose: black as a viscosigen (0–34%, m/m, η_rel_ = 1.0–3.6 cP). The solid lines represent a fit of the data to eq 4, indicating a linear dependency. The slopes of the viscosity effects on the normalized *k*_cat_ and *k*_cat_/K_m_ values are reported in Table 5.

**Figure 4:**
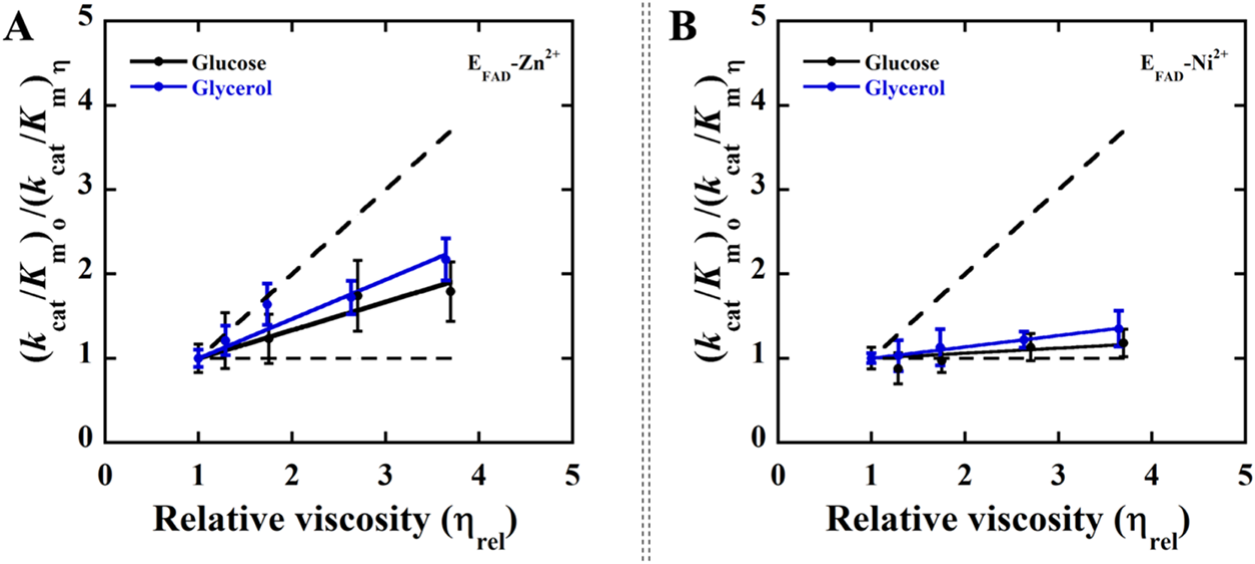
Effects of solvent viscosity on the *k*_cat_/*K*_m_ parameters with the alternate substrate D-malate. (A) Viscosity effects on the *k*_cat_/K_m_ parameter of E_FAD_-Zn^2+^. (B) Viscosity effects on the *k*_cat_/K_m_ parameter of E_FAD_-Ni^2+^. The dashed line with a slope of 1 and a slope of 0 describes a case in which the reaction is diffusion-controlled and not affected by diffusion, respectively. The enzyme activity assays were carried out using a Clark-type oxygen electrode in 25 mM NaPO_4_, 1 mM PMS, pH 7.4 thermostated at 25 °C, containing varying amounts of glycerol: blue (0–40%, m/m, η_rel_ = 1.0–3.5 cP) and glucose: black as a viscosigen (0–34%, m/m, η_rel_ = 1.0–3.6 cP). The solid lines report a fit of the data to eq 4 for linear dependency. The slopes of the viscosity effects on the normalized *k*_cat_ and *k*_cat_/K_m_ values are reported in Table 5.

**Table 5:** Kinetic Solvent Viscosity Effects on the Steady-State Kinetic Parameters of D-2-Hydroxyglutarate Dehydrogenase Enzyme Species^a^

	*k*_cat_		*k*_cat_/*K*_m_	
enzymes	glucose	glycerol	glucose	glycerol
E_FAD_-Zn^2+^	0.52 ± 0.04	0.32 ± 0.02	0.34 ± 0.04	0.47 ± 0.05
E_FAD_-Ni^2+^	0	0	0.06 ± 0.02	0.14 ± 0.01

a The enzyme activity assays were carried out using a Clark-type oxygen electrode in 25 mM NaPO_4_, 1 mM PMS, pH 7.4 thermostated at 25 °C, containing varying amounts of glycerol (0–40%, m/m, η_rel_ = 1.0–3.5 cP) or glucose as a viscosigen (0–34%, m/m, η_rel_ = 1.0–3.6 cP). The slopes from the viscosity effects were extrapolated after fitting the data to eq 4.

## Discussion

The *P. aeruginosa* D-2-hydroxyglutarate dehydrogenase is a recently characterized α-hydroxy acid dehydrogenase that converts D-2-hydroxyglutarate to 2-ketoglutarate.^46–50,58^ The enzyme requires FAD and Zn^2+^ for catalysis.^46–48,50,58^ Previous studies have proposed a mechanistic model for *P. aeruginosa* enzyme, implicating Zn^2+^ as the proton abstractor from C2 hydroxyl groups of the α-hydroxy acid substrate that initiates substrate oxidation and flavin reduction.^48,49^ Although Zn^2+^ is the native cofactor, the enzyme also retains catalytic activity in the presence of alternative metal ions such as Co^2+^, Mn^2+^, Ni^2+^, and Cd^2+^.^46,47,49,58,59^ Among these, Ni^2+^, despite its smaller ionic radius and higher electronegativity, can substitute for Zn^2+^ without compromising flavin integrity or the overall structural fold of the enzyme.^47,58,59^ The current study aimed to investigate the effects of Zn^2+^ substitution with the alternative metal ion Ni^2+^ on the kinetic mechanism of the enzyme. The mechanistic analysis demonstrates that the substitution of Zn^2+^ with Ni^2+^ significantly increases the rate of substrate dissociation and product release in D-2-hydroxyglutarate dehydrogenase. The study also revealed that the substitution of Zn^2+^ with Ni^2+^ alters the rate-limiting steps of the overall catalytic turnover of the enzyme. The overall catalytic turnover of E_FAD_-Ni^2+^ is fully limited by internal isomerization, with product release and hydride transfer no longer serving as the rate-determining step. In contrast, the rate of flavin reduction and product release controls the overall catalytic turnover of E_FAD_-Zn^2+^. The evidence supporting these conclusions is provided below.

### Substitution of Zn^2+^ with Ni^2+^ Results in a Faster Rate of Product Release in D-2-Hydroxyglutarate Dehydrogenase

Evidence to support this conclusion comes from the kinetic solvent viscosity effect on the steady-state kinetic parameters of E_FAD_-Ni^2+^ with D-malate. The viscosity plot of the normalized *k*_cat_ values as a function of the relative solvent viscosity in E_FAD_-Ni^2+^ exhibits a lack of viscosity effects on the *k*_cat_ values (Figure 3), consistent with the rate of oxaloacetate release *k*_5_ from the enzyme being significantly faster than the chemical step of catalysis *k*_3_.^72–75^ Alternatively, the slope of the *k*_cat_ viscosity plot can be used to calculate the oxaloacetate release (*k*_P‑rel_) for E_FAD_-Zn^2+^, which is 150 ± 80 s^–1^ using eq 5, and experimentally determined *k*_red_ and *k*_rev_ values (Table 4 and Figure 3).

5 $$\mathrm{slope}\left(m\right)=\frac{k_{\mathrm{red}}+k_{\mathrm{rev}}}{k_{\mathrm{red}}+k_{\mathrm{rev}}+k_{P-\mathrm{rel}}}$$

The rate of oxaloacetate release from the E_FAD_-Ni^2+^ enzyme is significantly faster than that of E_FAD_-Zn^2+^, likely due to the higher electronegativity of Ni^2+^ compared to Zn^2+^ (Table 6). The higher electronegativity of Ni^2+^ likely led to weaker interactions between Ni^2+^ and the oxaloacetate C1-carboxylate oxygen, which contribute to the faster rate of product release from the enzyme–product complex in the E_FAD_-Ni^2+^ compared to the E_FAD_-Zn^2+^.

**Table 6:** Physicochemical Properties of Transition Metal Ions^4,14^

metal ions	atomic^a^ weight^14^	ionic radius^b^ (Å)^10^	hydrated^b^ radius (Å)^10^	electronegativity^c^,^76^	atomic^d^ charge^77^
Zn^2+^	65.6	0.74	4.30	1.65	0.91
Ni^2+^	58.7	0.70	4.04	1.91	0.73

a Atomic weight derived from H. Zhang et al.^14^

b Ionic radius and hydrated radius derived from Nightingale.^10,14^

c Electronegetivity derived from Trivedi et al.^14,76^

d Atomic charge derived from VE. Jackson et al.^77^

### Substitution of Zn^2+^ with Ni^2+^ Decreases Substrate Stickiness in D-2-Hydroxyglutarate Dehydrogenase

Evidence supporting this conclusion comes from the reductive half-reaction and kinetic solvent viscosity effects using D-malate as a substrate for E_FAD_-Ni^2+^. The lack of viscosity effects on the *k*_cat_/*K*_m_ values of the E_FAD_-Ni^2+^ (Figure 4) suggests that dissociation of the substrate from the oxidized enzyme–substrate complex is significantly faster than the rate of chemical steps occurring within enzyme–substrate complexes belonging to *k*_cat_/*K*_m_, i.e., *k*_2_ is considerably larger than *k*_3_ (Scheme 2).^72–74^ Here, *k*_2_ dominates over *k*_3_, meaning that the substrate is less sticky and more likely to dissociate than to undergo conversion into a product. As the catalytic step (*k*_3_) in the E_FAD_-Ni^2+^ enzyme is negligible compared to the rate constant for substrate dissociation from the enzyme–substrate complex (*k*_2_), the substrate binding affinity (*K*_d_) is governed by the ratio of the rate of substrate dissociation (*k*_2_) to the rate of substrate association (*k*_1_) (Scheme 2). On the other hand, in E_FAD_-Zn^2+^, the linear pattern of the viscosity plots of the normalized *k*_cat_/*K*_m_ values as a function of the relative solvent viscosity yielded straight lines with a positive slope between 0 and 1 (Table 5), consistent with the capture of the substrate into enzyme–substrate complexes that yield products being partially limited by the rate of diffusion of the substrate (*k*_1_) into the active site of the enzyme.^73^ In this case, the slope will depend on the relative magnitude of all the kinetic rate constants that contribute to *k*_cat_/*K*_m_ except for *k*_1_.^73^ The slope of the *k*_cat_/*K*_m_ viscosity plot can be used to calculate the *k*_2_ value of E_FAD_-Zn^2+^, which is 80 ± 50 s^–1^ using eq 6,[Fn fn2] and experimentally determined *k*_3_ value (68 s^–1^).

6 $$\mathrm{slope}\left(m\right)=\frac{k_{3}}{k_{2}+k_{3}}$$

**Scheme 2:**
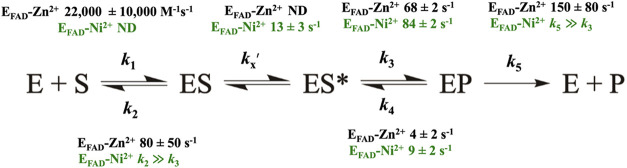
Microscopic Rate Constants of E_FAD_-Zn^2+^ (black) and E_FAD_-Ni^2+^ (green)^a^

a ND (black)—not determined, as the internal isomerization step is either absent or does not contribute to the rate-limiting process. ND (green)—not determined, as *k*_2_ is faster than *k*_3_.

For E_FAD_-Zn^2+^, the substrate can either dissociate from the enzyme or proceed to product formation at comparable rates (Scheme 2), making the *K*_d_ dependent on both steps (*K*_d_ = (*k*_2_ + *k*_3_)/*k*_1_), which results in a sticky substrate, one that binds efficiently to the enzyme such that its dissociation rate is slower than or comparable to the rate of chemical steps of catalysis. The higher electronegativity of Ni^2+^ than that of Zn^2+^ likely led to weaker interactions between Ni^2+^ and the D-malate C1-carboxylate and C2-hydroxyl oxygen, which decreases the substrate stickiness in E_FAD_-Ni^2+^. Alternatively, the smaller charge density of Ni^2+^ (charge-to-ionic radius ratio)^58^ may form a less stable Ni^2+^-D-malate C1-carboxylate and C2-hydroxyl complex, making the substrate less sticky in E_FAD_-Ni^2+^, which cannot be ruled out as a contributing factor. Additionally, differences in the ionic and hydrated radii of Ni^2+^ and Zn^2+^ (Table 6) likely change the interaction distance with the substrate in the active site, suggesting that electronegativity alone does not fully explain the observed effects.

### Product Release Is Not Rate-Limiting in the Overall Catalytic Turnover of the E_FAD_-Ni^2+^ Enzyme

Evidence supporting this conclusion comes from the reductive half-reaction, steady-state kinetics, and kinetic solvent viscosity effects with D-malate as a substrate for the E_FAD_-Ni^2+^. Notably, the E_FAD_-Ni^2+^ exhibits no viscosity effects on the *k*_cat_ parameter, indicating that the overall catalytic turnover is not limited by diffusional processes; i.e., product release is significantly faster and therefore not rate-limiting. For a reaction in which the overall turnover is entirely limited by the chemical step of catalysis (i.e., where *k*_cat_ = *k*_red_), an increase in solvent viscosity would not affect the *k*_cat_ value, resulting in a slope of zero in a normalized plot of *k*_cat_ versus relative solvent viscosity.^72,73,75^ Interestingly, the observed *k*_cat_ value of 10 s^–1^ for E_FAD_-Ni^2+^ was modestly but significantly lower than the corresponding *k*_red_ value of 84 s^–1^ for flavin reduction by D-malate (Tables 2 and 4). The discrepancy in the *k*_cat_ and *k*_red_ values indicates that an additional step, rather than the chemical step of hydride transfer, predominantly limits the overall turnover of E_FAD_-Ni^2+^. Previous KSVE and pL profile studies on E_FAD_-Zn^2+^ indicate that an internal isomerization of the enzyme–substrate (ES) complex precedes flavin reduction, such that hydride transfer is gated by conformational changes of the ES complex.^48^ Hence, a rate constant for an internal ES complex isomerization (*k*_*x*_′) was computed for E_FAD_-Ni^2+^ using the experimentally determined *k*_cat_, *k*_red_, and *k*_rev_ parameter values (Table 2) as shown in eq 7. The resulting estimated E_FAD_-Ni^2+^ *k*_*x*_′ value was 13 ± 2 s^–1^ (Scheme 2), which is comparable to the experimentally determined *k*_cat_ value of 10 s^–1^ for E_FAD_-Ni^2+^. The data suggest that an internal isomerization of the enzyme–substrate complex, rather than hydride transfer, predominantly limits the overall catalytic turnover of the E_FAD_-Ni^2+^ enzyme, and the rate of hydride transfer, *k*_red_, contributes only marginally to the *k*_cat_ parameter value (Scheme 2).^72,73,78^ Hence, internal ES complex isomerization is the primary rate-limiting step in the E_FAD_-Ni^2+^ enzyme.

7 $$k_{\mathrm{cat}}=\frac{k_{\mathrm{red}}k_{x^{\prime }}}{k_{\mathrm{red}}+k_{\mathrm{rev}}+k_{{x^{\prime }}^{\;}}}$$

Instead, the overall turnover of the E_FAD_-Zn^2+^ enzyme is limited by both flavin reduction (68 s^–1^) and product release (150 s^–1^), as indicated by the linear solvent viscosity plot with a positive slope (Figure 3A and Table 5). The internal isomerization of the ES complex that gates flavin reduction does not contribute to the rate-limiting process in the E_FAD_-Zn^2+^ enzyme, due to the slower product release step (Scheme 2). The observation that internal isomerization is predominantly rate-limiting in the E_FAD_-Ni^2+^ enzyme is likely due to the smaller ionic radius and atomic weight of Ni^2+^ compared to those of Zn^2+^ (Table 6). The smaller size of Ni^2+^ necessitates a specific orientation of D-malate within the active site of E_FAD_-Ni^2+^, which likely slows down D-malate binding and may translate into a slower rate of the internal isomerization step that precedes flavin reduction. However, more mechanistic evidence is required to fully explain the effects of metal electronic properties.

### Replacement of Zn^2+^ with Ni^2+^ Results in Minimal Changes to the Rate of Flavin Reduction in D-2-Hydroxyglutarate Dehydrogenase

This conclusion is drawn by comparing the reductive half-reactions of E_FAD_-Ni^2+^ and E_FAD_-Zn^2+^ with D-malate at pH 7.4 and 25 °C. The first-order rate constant for flavin reduction at a saturating D-malate concentration, *k*_red,_ which reports the hydride transfer reaction, was almost similar in the E_FAD_-Ni^2+^ (*k*_red_ = 84 s^–1^) compared to the native Zn^2+^ enzyme (*k*_red_ = 68 s^–1^). The metal ion Ni^2+^ exhibited flavin reduction rates comparable to those of Zn^2+^, suggesting that their electronic and coordination properties do not significantly perturb the positioning of the C2 hydroxyl group of D-malate relative to the flavin. The fully occupied 3d^10^ configuration of Zn^2+^ likely limits ligand dynamics in the enzyme, resulting in a relatively rigid active site. The electronic configurations of Ni^2+^ (3d^8^) likely contribute to metal–ligand interactions that maintain a similar substrate C2 hydroxyl group orientation for hydride transfer, compared to Zn^2+^, due to the presence of the 3d configuration. A previous study on human carbonic anhydrase II demonstrated that replacing Zn^2+^ with Ni^2+^ reduces enzymatic activity to ∼2% of the native Zn^2+^ enzyme.^8^ The loss in activity was attributed to Ni^2+^ adopting an octahedral coordination geometry (vs tetrahedral for Zn^2+^), which impedes CO_2_ binding and disrupts the active-site water network essential for proton transfer.^8^ In contrast, D-2-hydroxyglutarate dehydrogenase appears to be more tolerant to metal substitution. Despite the structural and electronic difference between Zn^2+^ and Ni^2+^, the E_FAD_-Ni^2+^ enzyme retains its catalytic function without compromising the rate of flavin reduction.

### Substituting Zn^2+^ with Ni^2+^ Exerts a Negligible Effect on the Catalytic Turnover and Efficiency of D-2-Hydroxyglutarate Dehydrogenase

Evidence supporting this conclusion comes from steady-state kinetic comparisons of E_FAD_-Ni^2+^ with E_FAD_-Zn^2+^ using D-malate or D-2-hydroxyglutarate. E_FAD_-Ni^2+^ catalyzes the oxidation of D-malate to oxaloacetate with only a 4-fold decrease in *k*_cat_ value and a 2.4-fold increase in *k*_cat_/*K*_m_ value relative to E_FAD_-Zn^2+^, suggesting that substitution of Zn^2+^ with Ni^2+^ has a minimal impact on D-malate oxidation (Table 2). A similar trend was observed with the physiological substrate D2HG, for which the *k*_cat_ value decreased by only 2-fold and the *k*_cat_/*K*_m_ value decreased by 1.5-fold upon metal substitution (Table 3). Comparison of the two substrates revealed no substantial changes in the *k*_cat_ values for either E_FAD_-Zn^2+^ or E_FAD_-Ni^2+^, whereas *k*_at_/*K*_m_ increased 20- and 5-fold, respectively, when D-malate was replaced with D2HG (Tables 2 and 3). The simplest rationale for the similar turnover rates observed with four- and five-carbon substrates is a conserved binding geometry in which the substrate C1 carboxylate and C2 hydroxyl oxygen atoms engage in bidentate coordination with the active-site metal ion.^47^ While differences in catalytic efficiency likely arise from distinct electrostatic interactions involving the C5 carboxylate in the five-carbon substrate with the active-site residue.

Nevertheless, despite the similarity in the *k*_cat_/*K*_m_ values, their underlying canonical expressions differ in terms of E_FAD_-Zn^2+^ and E_FAD_-Ni^2+^. The general canonical expression for the second-order rate constant for substrate capture, *k*_cat_/*K*_m_, is given by eq 8. At saturating concentration of PMS, which will force the E_FADred_-D-malate species to partition forward rather than reverting through the *k*_4_ step, eq 9 simplifies to eq 10, establishing that the second-order rate constant *k*_cat_/*K*_m_ comprises both the chemical step of substrate oxidation (*k*_3_) and the rate constant for the association (*k*_1_) and dissociation (*k*_2_) of the substrate to and from the enzyme E_FAD_-Zn^2+^. The *k*_1_ value for E_FAD_-Zn^2+^ was calculated to be 22,000 ± 10,000 M^–1^ s^–1^ (Scheme 2), using eq 10, and the estimated *k*_2_ value from eq 6, as previously shown. For E_FAD_-Ni^2+^, the lack of solvent viscosity effect on the *k*_cat_/*K*_m_ value is consistent with *k*_2_ ≫ *k*_3_, resulting in the *k*_cat_/*K*_m_ value being the ratio of the rate constant that defines the chemical step of catalysis to the dissociation constant that defines substrate binding (*K*_d_), as illustrated in eq 11. The calculated *k*_cat_/*K*_m_ value for E_FAD_-Ni^2+^ is 21,000 ± 5000 M^–1^s^–1^ using eq 11, which is closely similar to the experimentally observed value (Table 2). Moreover, for E_FAD_-Ni^2+^, *K*_d_ is defined as *k*_2_/*k*_1_. If this relationship is incorporated into eq 11, it becomes evident that neither *k*_1_ nor *k*_2_ can be independently determined using experimentally determined *k*_cat_/*K*_m_ and *k*_3_ values for E_FAD_-Ni^2+^ for making a comparison with E_FAD_-Zn^2+^ (Scheme 2).

8 $$k_{\mathrm{cat}}/K_{m}=\frac{k_{1}k_{3}k_{5}}{k_{2}{k_{4}+k}_{2}k_{5}+k_{3}k_{5}}$$

9 $$k_{\mathrm{cat}}/K_{m}=\frac{k_{1}k_{3}}{{(k}_{2}k_{4}/k_{5})+k_{2}+k_{3}}$$

10 $$k_{\mathrm{cat}}/K_{m}=\frac{k_{1}k_{3}}{k_{2}+k_{3}}$$

11 $$k_{\mathrm{cat}}/K_{m}=\frac{k_{3}}{K_{d}}$$

## Conclusions

In conclusion, this study highlights the crucial role of metal ion substitution in regulating the kinetic mechanism of *P. aeruginosa* D-2-hydroxyglutarate dehydrogenase. Replacing Zn^2+^ with Ni^2+^ significantly accelerates substrate dissociation and product release, likely due to the higher electronegativity of Ni^2+^. The higher electronegativity of Ni^2+^ likely led to repulsion with the D-malate C1 carboxylate and C2 hydroxyl oxygen, which contribute to the faster rate of substrate dissociation from the enzyme–substrate complex and product release from the enzyme–product complex in the E_FAD_-Ni^2+^ enzyme. The smaller ionic and hydrated radii of Ni^2+^ likely modify the interaction distance with the substrate, providing a more complete physicochemical rationale for the decreased substrate and product “stickiness” in the Ni^2+^ enzyme. The study also revealed that the substitution of Zn^2+^ with Ni^2+^ alters the rate-limiting steps of the overall catalytic turnover of the enzyme. In the Zn^2+^-bound enzyme, flavin reduction and product release are the primary determinants of overall catalytic turnover, with an internal isomerization step likely masked by slower product release. In contrast, for the Ni^2+^-substituted enzyme, turnover is governed by internal isomerization with product release and hydride transfer no longer rate-limiting. The internal isomerization that is predominantly rate-limiting in the E_FAD_-Ni^2+^ enzyme is likely due to the smaller ionic radius of Ni^2+^ compared to that of Zn^2+^. The smaller ionic radius of Ni^2+^ requires the spatial orientation of D-malate within the active site to enable efficient hydride transfer to the enzyme-bound FAD. Overall, the findings demonstrate that the physicochemical properties of metal ion cofactors, such as electronegativity, ionic radius, hydrated radius, and charge density, can combinedly influence the kinetic mechanism of *P. aeruginosa* D-2-hydroxyglutarate dehydrogenase.

## Supplementary Material



## Data Availability

All data are contained within the manuscript, except that reported in the Supporting Information.

## Footnotes

1 The two kinetic constants, *K*_d,_ and *K*_m_, represent different kinetic steps in the enzyme’s catalytic cycle. The interpretation of substrate “stickiness” is based on *k*_2_ (substrate dissociation), not *K*_m_, as *K*_m_ is a composite kinetic constant and does not directly report on substrate binding affinity. This parameter reflects the interplay of multiple steps in the enzymatic cycle, including substrate association and dissociation, and the chemical step of catalysis, in both forward and reverse directions, rather than purely binding affinity. Thus, we have not directly compared the *K*_m_ values between different enzyme species. The derivations of *K*_m_ for both enzyme forms are provided below to illustrate the kinetic complexity. For E_FAD_-Ni^2+^, $k_{\mathrm{cat}}=\frac{k_{3}k_{x^{\prime }}}{k_{3}+k_{4}+k_{x^{\prime }}}$ , and $k_{\mathrm{cat}}/K_{m}=\frac{k_{1}k_{3}}{k_{2}}$ . The ratio of *k*_cat_, and *k*_cat_/*K*_m_ is equal to *K*_m_, which is equal to $\frac{k_{3}k_{x^{\prime }}}{k_{3}+k_{4}+k_{x^{\prime }}}\cdot \frac{k_{2}}{k_{1}k_{3}}$ . The *k*_3_ parameter is canceled out from both numerator and denominator, resulting *K*_m_ is equal to $\frac{k_{2}k_{x^{\prime }}}{{k_{1}k}_{3}+{k_{1}k_{4}+k_{1}k}_{x^{\prime }}}$ . However, for E_FAD_-Zn^2+^, $k_{\mathrm{cat}}=\frac{k_{3}k_{x^{\prime }}}{k_{3}+k_{4}+k_{x^{\prime }}}$ , and $k_{\mathrm{cat}}/K_{m}=\frac{k_{1}k_{3}}{k_{2}+k_{3}}$ . The ratio of *k*_cat_, and *k*_cat_/*K*_m_ is equal to *K*_m_, which is equal to $\frac{k_{3}k_{x^{\prime }}}{k_{3}+k_{4}+k_{x^{\prime }}}\cdot \frac{k_{2}+k_{3}}{k_{1}k_{3}}$ . The *k*_3_ parameter is canceled out from both numerator and denominator, resulting *K*_m_ is equal to $\frac{k_{2}k_{x^{\prime }}+k_{3}k_{x^{\prime }}}{{k_{1}k}_{3}+{k_{1}k_{4}+k_{1}k}_{x^{\prime }}}$ .

2
Eq 6 is derived from the general analytical description for the slope of a plot of (*k*_cat_/*K*_m_)_o_/(*k*_cat_/*K*_m_)_η_ vs η_rel_ for reversible enzymatic reactions, $\mathrm{slope}=\frac{k_{3}k_{5}}{k_{2}{k_{4}+k}_{2}k_{5}+k_{3}k_{5}}$ by dividing both numerator and denominator by *k*_5_. Here, *k*_2_ *k*_4_/*k*_5_ is negligible as *k*_5_ value 150 ± 80 s^–1^, which is 40-times larger than *k*_4_ value (4 ± 2 s^–1^) for E_FAD_-Zn^2+^.

## Notes

SDS–PAGE of D-2-hydroxyglutarate dehydrogenase E_FAD_-Zn^2+^ and E_FAD_-Ni^2+^ species; steady-state kinetics of D-2-hydroxyglutarate dehydrogenase E_FAD_-Zn^2+^ and E_FAD_-Ni^2+^ species with varying D-malate and fixed saturating PMS (PDF)

## ∥

Department of Chemistry, Princeton University, Frick Chemistry Laboratory, Corner of Washington Road and Scholar Way, Princeton, 08544, New Jersey, Unites States

## Abbreviations

E_FAD_-apo: *Pseudomonas aeruginosa* metallo-apoenzyme
E_FAD_-M^2+^: *Pseudomonas aeruginosa* D-2-hydroxyglutarate dehydrogenase loaded with metal ions (M^2+^ = Zn^2+^, or Ni^2+^)
D2HG: D-2-hydroxyglutarate
D2HGDH: D-2-hydroxyglutarate dehydrogenase
ICP-MS: inductively coupled plasma-mass spectrometry
EDTA: ethylenediamine tetra acetic
