# Supplementary material for: Substitution of Zn2+ with Ni2+ Alters Kinetic Steps in D‑2-Hydroxyglutarate Dehydrogenase from Pseudomonas aeruginosa PAO1
Source: ACS Omega. 2025 Dec 15;10(51):63380–91. doi: 10.1021/acsomega.5c09933 (PMC12756731; doi:10.1021/acsomega.5c09933)
Supplement: Supplementary file 1 [file ao5c09933_si_001.pdf]

Supporting Information: **Substitution of Zn^{2+} with Ni^{2+} alters kinetic steps in D-2-hydroxyglutarate dehydrogenase from *Pseudomonas aeruginosa* PAO1**

Bilkis Mehrin Moni[1], Junkai Yang[1], Joanna Afokai Quaye[1], and Giovanni Gadda[1,2,3,*]

[1]Departments of Chemistry, [2]Biology, and [3]The Center for Diagnostics and Therapeutics, Georgia State University, Atlanta, GA 30302-3965, U.S.A.

Corresponding author

*To whom correspondence should be addressed: Giovanni Gadda, Department of Chemistry, Georgia State University, P.O. Box 3965, Atlanta, GA 30302-3965, U.S.A.

Phone: (404) 413-5537

FAX: (404) 413-5505

EMAIL: ggadda@gsu.edu

Keywords

D-2-hydroxyglutarate dehydrogenase, *Pseudomonas aeruginosa,* metal substitution, catalytic activity, product release, substrate dissociation.

Figure S1

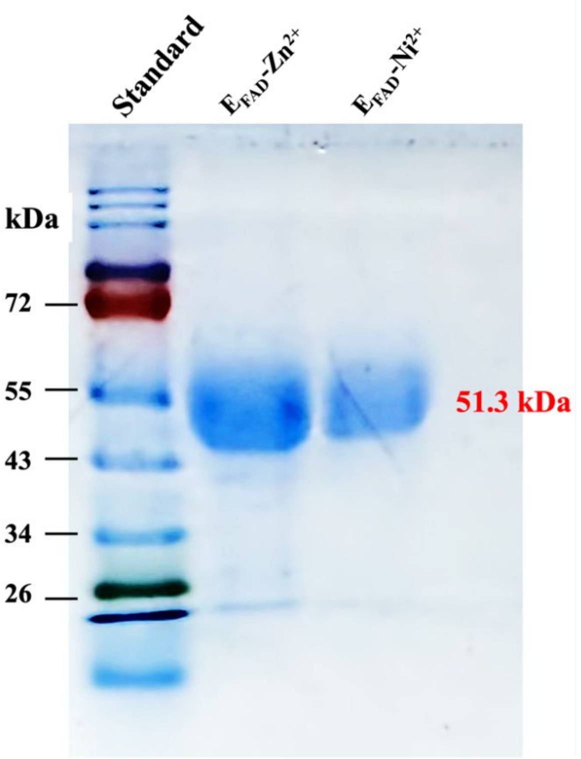

Figure S1: SDS-PAGE of D-2-hydroxyglutarate dehydrogenase E_{FAD}-Zn^{2+} and E_{FAD}-Ni^{2+} species. The recombinant protein was purified to homogeneity as judged by the SDS-PAGE analysis, with a single prominent band corresponding to the expected molecular weight of ~51.3 kDa.

Table S1

Table S1 : *Steady-state kinetics of D-2-hydroxyglutarate dehydrogenase E_{FAD}-Zn^{2+} and E_{FAD}-Ni^{2+} species with varying D-malate and fixed saturating PMS

Enzymes	[PMS] (mM)	k_{cat} (s^{-1})	k_{cat}/K_m ($M^{-1}s^{-1}$)
**E_{FAD}-Ni^{2+}**	0.1	7 ± 1	32,000 ± 5000
	1	12 ± 1	28,000 ± 5000
	2	12 ± 1	32,000 ± 5000
**E_{FAD}-Zn^{2+}**	0.1	40 ± 2	20,000 ± 4000
	1	40 ± 2	20,000 ± 3000
	2	41 ± 2	20,000 ± 5000

* The kinetic parameters were determined with fixed PMS concentrations at 0.1, 1, and 2 mM in 25 mM $NaPO_4$, pH 7.4, and 25 ℃, using steady-state kinetics. The D-malate concentrations were between 1-60 mM for E_{FAD}-Zn^{2+} or 0.2-40 mM for E_{FAD}-Zn^{2+}.
